# Function, Role, and Clinical Application of MicroRNAs in Vascular Aging

**DOI:** 10.1155/2016/6021394

**Published:** 2016-12-21

**Authors:** Xiao Lin, Jun-Kun Zhan, Yan-Jiao Wang, Pan Tan, Yi-Yin Chen, Hui-Qian Deng, You-Shuo Liu

**Affiliations:** Department of Geriatrics, The Second Xiangya Hospital, Central South University, Changsha, Hunan 410011, China

## Abstract

Vascular aging, a specific type of organic aging, is related to age-dependent changes in the vasculature, including atherosclerotic plaques, arterial stiffness, fibrosis, and increased intimal thickening. Vascular aging could influence the threshold, process, and severity of various cardiovascular diseases, thus making it one of the most important risk factors in the high mortality of cardiovascular diseases. As endothelial cells (ECs) and vascular smooth muscle cells (VSMCs) are the main cell biological basis of these pathology changes of the vasculature, the structure and function of ECs and VSMCs play a key role in vascular aging. MicroRNAs (miRNAs), small noncoding RNAs, have been shown to regulate the expression of multiple messenger RNAs (mRNAs) posttranscriptionally, contributing to many crucial aspects of cell biology. Recently, miRNAs with functions associated with aging or aging-related diseases have been studied. In this review, we will summarize the reported role of miRNAs in the process of vascular aging with special emphasis on EC and VSMC functions. In addition, the potential application of miRNAs to clinical practice for the diagnosis and treatment of cardiovascular diseases will also be discussed.

## 1. Introduction

Aging is a multifactorial process characterized by a progressive loss of physiological integrity and functionality, which increases mortality and susceptibility to diseases, including cardiovascular diseases, diabetes, osteoporosis, immunological diseases, various neurodegenerative diseases, and cancer [1–3]. Vascular aging is a specific type of organic aging playing a key role in the process of overall aging. Vascular aging is one of the most important risk factors in the high mortality of cardiovascular diseases and could influence the threshold, process, and severity of various cardiovascular diseases. Vascular aging is tightly linked to alterations in the biological functions and structural properties of the vascular wall, mainly including endothelial cells (ECs) and vascular smooth muscle cells (VSMCs). ECs, the inner layer of blood vessels, on the one hand, serve as a barrier between the blood stream and vessel and, on the other hand, regulate many aspects of vessel function, such as the control of vasodilation and vasoconstriction, inhibition of the adhesion of leukocytes and platelets to prevent blood coagulation, and suppression of vessel wall hypertrophy by inhibiting VSMC proliferation [4]. Furthermore, structural damage and dysfunction of ECs, such as senescence, apoptosis, proliferation, and inflammation, are closely associated with vascular aging. VSMCs, the main cells of the media vessel wall, can control blood flow by contracting or relaxing in response to external stimuli and play an important role in vascular pathologies. With increasing age, VSMCs are thought to undergo a phenotypic change from the quiescent, contractile phenotype to a synthetic phenotype. This synthetic phenotype is capable of migration into the intima and subsequent proliferation and extracellular matrix (ECM) synthesis, which in turn affects vascular function and disease outcome in the elderly [5]. Because vessels serve as transportation tools, they could supply nutrients, oxygen, and active substances and remove wastes or by-products and carbon dioxide produced in tissues [6]. Thus, vessels are critical to maintaining physiological homeostasis in vivo; as William Osler said, “a man is just as old as his arteries” [7]. Therefore, a better understanding of vascular physiological and functional changes with aging is necessary and crucial to combat cardiovascular diseases in the future.

MicroRNAs (miRNAs), which are small noncoding RNAs, are approximately 18–25 nucleotides long. miRNA genes are mainly transcribed by RNA polymerase. They are initially transcribed as large precursors, called primary miRNAs (pri-miRNAs). Pri-miRNAs are then processed by the RNase-III enzymes Drosha and Dicer to generate mature miRNA products. Recently, studies have shown that miRNAs could cause target mRNA degradation at the posttranscriptional level and/or suppress the translation of mRNA into protein via interaction with the 3′-untranslated region (3′UTR) of target mRNA by complementary base pairing [8]. So far, over 1000 miRNAs have been found in human cells. Each miRNA might target several genes and different miRNAs might target the same gene. As negative regulators of gene regulation, miRNAs contribute to many essential physiological and pathophysiological processes in humans, including differentiation, proliferation, apoptosis, migration, homeostasis, and various diseases [9, 10]. Therefore, it is not surprising that miRNAs are also involved in vascular aging and age-related diseases because of their multiple biological functions [11, 12].

## 2. miRNAs and Vascular Aging

Increasing evidence has shown that aging-associated physiological and functional disorders are associated with alterations in miRNAs, suggesting that miRNAs are novel cellular senescence regulators [59, 60]. Vascular aging is tightly linked to alterations in the biomechanical and structural properties of the vascular wall, including ECs and VSMCs dysfunction or apoptosis as well as increased arterial stiffness [11]. Until now, however, only the functions of a few miRNAs have been associated with cell dysfunction and/or vascular aging. In this section, we will discuss the role of miRNAs in the progression of vascular aging.

### 2.1. miRNAs and Endothelial Function

The vascular endothelium, a thin layer of ECs that lines the inner surface of blood vessels, is a critical interface between blood and all tissues. When the endothelium is exposed to various stimuli, such as hypoxia, proinflammatory cytokines, oxidative stress, hypertension, hyperglycemia, shear stress, aging, or injury, the function of ECs will be influenced, which is related to the proliferation, apoptosis, migration, senescence, angiogenesis, and inflammation of ECs [61]. Here, we will focus on individual miRNAs associated with endothelial functions (Table 1).

#### 2.1.1. Influence of miRNAs on EC Apoptosis

EC apoptosis plays a vital role in the initiation and progression of atherosclerotic. In addition, EC apoptosis is responsible for plaque instability because EC death can predispose to arterial thrombosis, which could cause acute coronary occlusion and sudden death [62]. Accumulating evidence has indicated that miRNAs act as critical regulators to participate in EC apoptosis.

Several miRNAs are involved in the regulatory mechanisms of cellular apoptosis of ECs. Some are antiapoptotic miRNAs while others have proapoptotic effects. miR-126 was the most abundant miRNA in apoptotic bodies derived from ECs. It induced CXCL12 expression by targeting RGS16 and protected mice from atherosclerosis in a miR-126-dependent manner [63]. Recently, Chen et al. also demonstrated that miR-126 inhibits vascular ECs apoptosis through targeting PI3K/Akt signaling pathway [15]. miR-495 targets CCL2, significantly promoting human umbilical vein endothelial cell (HUVEC) proliferation and inhibiting apoptosis by affecting the expression of cleaved caspase-3 [19]. In addition, miR-19b plays a key role in the attenuation of TNF-*α*-induced EC apoptosis, and this function is closely linked to the Apaf1/caspase-7-dependent pathway [22]. Nevertheless, miR-132 promoted apoptosis of HUVEC induced by TNF-*α* and inhibited its proliferation, viability, and migration by inhibiting SIRT1 [23].

Oxidatively modified low density lipoprotein (Ox-LDL) is a major risk factor in the development of atherosclerosis. miR-365 and miR-US25-1 exerted a proapoptotic function in ox-LDL treated ECs by targeting the inhibition of Bcl-2 and BRCC 3 expression, respectively [26, 27]. Another miRNA, named miR-26a, was sufficient to reverse ox-LDL-induced apoptosis; the underlying mechanisms likely involved repression of TRPC6 and the associated downstream apoptotic pathway [14]. Furthermore, the let-7 family was found to be related to atherosclerosis and coronary artery diseases and is highly expressed in ECs. The inhibitory effects of let-7a and let-7b on ox-LDL induced EC apoptosis and dysfunction are partly obtained through the LOX-1/ROS/p38MAPK/NF-*κ*B signaling pathway and the LOX-1/ROS/PKB/eNOS pathway [20]. Meanwhile, let-7g negatively regulated apoptosis in ECs by targeting caspase-3 expression [21]. In addition, miR-221/222 could partly alleviate apoptotic cell death mediated by ox-LDL through the suppression of Ets-1 and its downstream target, p21 [18].

Both intra- and extracellular conditions, such as shear stress, oxidative stress, hyperglycemia, and withdrawal, have a major effect on miRNA expression in EC functions, and the molecular mechanisms involved have been extensively studied [13, 16, 17, 24]. miR-21 targets PTEN and attenuates endothelial apoptosis by regulating Akt phosphorylation, eNOS phosphorylation, and NO production in human ECs [13]. G*α*12 protects HUVEC from serum withdrawal-induced apoptosis by retaining miR-155 expression [17]. In diabetes patients, miR-130a inhibits the JNK pathway by targeting MAP3K12, contributing to its antiapoptotic effect and the maintenance of endothelial progenitor cell (EPC) function under high glucose conditions [16]. Other miRNAs have a proapoptotic effect on ECs. For example, miR-200c is upregulated by oxidative stress and induces EC apoptosis and senescence via ZEB1 inhibition [24]. Moreover, platelet-released miR-223 promotes advanced glycation end product- (AGE-) induced vascular EC apoptosis via targeting of IGF-1 [25].

#### 2.1.2. Functions of miRNAs in EC Senescence

Senescence is associated with the cellular response to various environmental stressors and damage, which is defined as permanent cell cycle arrest. Senescent ECs are important in atherosclerosis and other age-related diseases [64]. An EC often undergoes both replicative and stress-induced presenescence. The function of miRNAs involved in the regulatory mechanisms of ECs senescence has been investigated. During replicative senescence of ECs, miR-22 could accelerate the process of aging by down regulating Vasohibin-1 [28]. However, miR-92a, a component of the miR-17-92 cluster, is highly expressed in young ECs. Rippe et al. reported that senescence of human ECs is associated with the reduced expression of miR-92a [32]. In the progress of stress-induced presenescence of ECs, miR-221 promotes senescence of human arterial ECs by inhibiting NO production and activating NF-*κ*B signaling in human ECs [32]. Increased expression of miR-200c by ROS could induce the cellular senescence target zinc finger E-box-binding homeobox 1 (ZEB1) [24].

SIRT1 is a longevity gene that protects cells against oxidative and genotoxic stress. Recent studies have indicated that miR-34a is highly expressed in ECs. miR-34a expression is increased in senescent HUVECs and induces HUVEC senescence through the suppression of SIRT1 [29]. Two other miRNAs, miR-217 and miR-146a, promote senescence with a reduction of SIRT1 in ECs [30, 31]. On the contrary, let-7g has the effect of reducing EC senescence by increasing SIRT1 protein levels [33].

#### 2.1.3. miRNAs and EC Proliferation

EC proliferation and viability are critical in the process of promoting endothelial healing and improving vascular function. Numerous lines of evidence support the involvement of miRNAs in EC proliferation. It has been reported that miR-495 significantly promoted HUVEC proliferation by directly targeting CCL2 [19]. Feng et al. demonstrated that miR-487b enhanced cell proliferation and migration in HUVECs through regulating THBS1 [36]. Apart from the influence on EC apoptosis, endothelial miR-126-5p could also promote the proliferation of ECs through suppression of the Notch1 inhibitor delta-like 1 homologue (Dlk1), thereby preventing the formation of atherosclerotic lesions [35]. Another highly expressed miRNA in endothelium is miR-29a, which was able to accelerate G1 to S cell cycle transition in HUVECs and enforce the expression of miR-29a in endothelium, remarkably promoting cell proliferation and angiogenesis via the targeting of HBP1 [34].

However, some miRNAs also exist that inhibit the proliferation of ECs. Both miR-34a and miR-92a are upregulated in ECs during aging, inhibiting cell proliferation and migration by targeting SIRT1 [29, 39]. Moreover, miR-21 can enhance the rapamycin-induced inhibition of endothelial proliferation by targeting RhoB [37]. In addition, miR-101 can induce cell cycle arrest at the G1/S transition and suppress mTOR expression and EC proliferation induced by laminar shear stress [40]. HUVEC proliferation is significantly inhibited by miR-125a and miR-24 via regulation of the expression of Bcl-2 and Sp1, respectively [38, 41].

#### 2.1.4. Effects of miRNAs on Endothelial Angiogenesis

Angiogenesis is the process of new blood vessel and capillary network formation in the body, which is essential for recovery after cardiac and skeletal muscle injury or ischemia. Aged individuals, however, appear to have impaired physiological angiogenesis and are at higher risk of processes associated with pathological vessel formation, whereas ECs play a crucial role in the initiation of angiogenesis and the formation of early vascular structures [65]. A large number of miRNAs are responsible for angiogenesis and are expressed in ECs [43, 49–51, 66]. Wang et al. reported that miR-126, the endothelial specific miRNA, enhances the proangiogenic actions of VEGF and FGF and promotes blood vessel formation by repressing the expression of Spred-1, an intracellular inhibitor of angiogenic signaling [43]. Besides, members of the miRNA-17-92 cluster also exhibit a cell-intrinsic antiangiogenic function in ECs [42, 66, 67]. For example, pre-miR-92a treatment improves HUVEC viability and preserves angiogenic capacity under oxidative stress, at least partially through the downregulation of PTEN expression [42]. Another study reported that miR-92a was identified as a negative regulator of angiogenesis by targeting the A5 integrin subunit (ITGA5) [67]. The contradictory results between the two studies might be attributed to the different functions of their different target proteins. Furthermore, miR-20a, another component of the miR-17–92 cluster, acts in a feedback loop to repress the expression of MKK3 and to negatively regulate p38 pathway-mediated VEGF-induced ECs migration and angiogenesis [45].

Other important miRNAs involved in angiogenesis regulation are the so-called antiangiogenic miRNAs, which include miR-221/222, miR-223, miR-206, miR-15a, miR-214, miR-21, miR-106b, miR-129-1, miR-133, miR-29c, miR-217, and miR-351. Poliseno et al. proved that miR-221/222 and miR-223 are antiangiogenic factors and that they affect the expression of the c-Kit receptor and *β*1 integrin in ECs, respectively [49, 50]. The signal transducer and activator of transcription 3 (STAT3) signaling pathway was regarded as a target for the prevention of atherosclerosis or other cardiovascular diseases. Previous studies showed that both miR-351 and miR-106b were upregulated in atherosclerotic mice and exerted an antiangiogenic effect in ECs by targeting STAT3 in vitro [47, 51]. Other miRNAs, such as miR-214, miR-21, and miR-15a, reduce angiogenesis of HUVEC by directly targeting XBP1, RhoB, and FGF2 and VEGF, respectively [44, 46, 48]. However, some miRNAs influence angiogenesis by affecting other functions of ECs. For example, miR-129-1 and miR-133 modulate angiogenesis by suppressing the proliferation rate, cell viability, and migration activity of HUVECs in vitro by targeting VEGFR2 and FGFR1, respectively [68]. Moreover, miR-29c plays a significant role in regulating angiogenic properties of HUVECs through the IGF-1/PI3K/AKT signaling pathway [69].

#### 2.1.5. miRNAs Associated with Endothelial Inflammation

ECs, activated by shear stress, lipopolysaccharides, or cytokines, can modulate the expression of adhesion molecules and chemokines, leukocytes rolling over the endothelium and adhesion to vessels [70], which are stimulators of inflammation. Inflammation is associated with the development and progression of age-related conditions and they make individuals, especially the aged, more susceptible to cardiovascular diseases. Moreover, inflammatory mediators also play a fundamental role in the initiation, progression, and eventual rupture of atherosclerotic plaques and could therefore accelerate vascular aging [71]. Recent reports have shown that miRNAs can control vascular inflammation by controlling leukocyte activation and infiltration through the vascular wall [72]. Loyer et al. reported that miR-92a acts as a proinflammatory regulator in ECs by activating inflammatory cytokines and chemokines and promoting monocyte adhesion [53]. Zhou et al. showed that miR-21 suppresses the translation of peroxisome proliferator-activated receptor *α* (PPAR*α*) mRNA, promoting endothelial inflammation by inducing the expression of vascular cell adhesion protein 1 (VCAM-1) and C–C motif chemokine 2 (CCL2) by increasing the activity of the transcription factor AP-1 [52].

Other important miRNAs associated with inflammation could inhibit endothelial inflammation. Harris et al. found that the inhibition of miR-126 increases proinflammatory TNF-*α* expression, which activates NF-*κ*B and interferon regulatory factor 1 and finally induces the expression of VCAM-1 and the adhesion of leukocytes to ECs [55]. The systemic delivery of miR-181b also attenuates atherosclerosis by targeting NF-*κ*B signaling in ECs [57]. miR-663, one of the oscillatory shear-sensitive miRNAs in HUVECs, is involved in oscillatory shear stress-induced cellular inflammation by regulating the potential targets of SLC7A5 and NAV2 [58]. miR-155 inhibits angiotensin II- (Ang II-) induced inflammation, migration, and apoptosis in HUVECs by targeting the Ang II type 1 receptor [56]. miR-30-5p acts in an anti-inflammatory manner in ECs induced by KLF2 and shear stress by impairing the expression of Ang2 and inflammatory cell-cell adhesion molecules [54]. Let-7g decreases EC inflammation and monocyte adhesion and increases angiogenesis via the TGF-*β* pathway [33].

**Figure 1:**
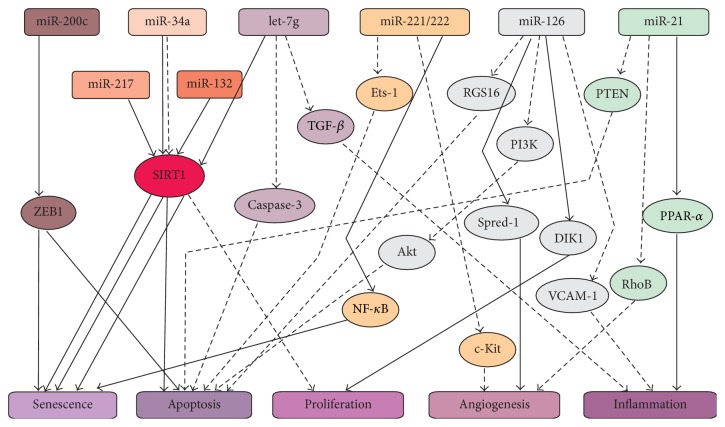
Network system of several important miRNAs regulating the function of ECs. The picture shows that SIRT1 is an important gene in the regulation of EC function. miR-34a, miR-217, miR-132, and let-7g are targets of SIRT1. Other miRNAs, such as miR-221/222, miR-126, and miR-21, participate in the function of ECs via targeting different genes. They can promote or inhibit the functions (senescence, apoptosis, proliferation, angiogenesis, and inflammation) of ECs. “—” indicates promotion effects; “—” denotes inhibition effects.

Several important miRNAs regulate different kinds of EC functions among those that participate in the functional regulation of ECs. For example, miRNA-126 can inhibit apoptosis in ECs via the PI3K/AKT signaling pathway [15]. Meanwhile, it also plays a role in promoting angiogenesis and inflammation in ECs [43, 55]. In addition, miR-221/222 is also involved in the regulation of apoptosis, senescence, and angiogenesis in ECs [18, 50]. Upon summarizing numerous previous studies, it is not difficult to conclude that the SIRT1 gene, initially identified as a longevity gene, plays an important role in the regulation of ECs function. On the one hand, SIRT1 can be regarded as a regulatory target of multiple miRNAs, such as miR-34a, miR-221/222, miR-217, miR-132, and let-7g; on the other hand, it is involved in regulating multiple functions of ECs, such as senescence, apoptosis, and proliferation.  Figure 1 shows the network of important miRNAs regulating the function of ECs.

### 2.2. miRNAs and VSMCs Function

**Figure 2:**
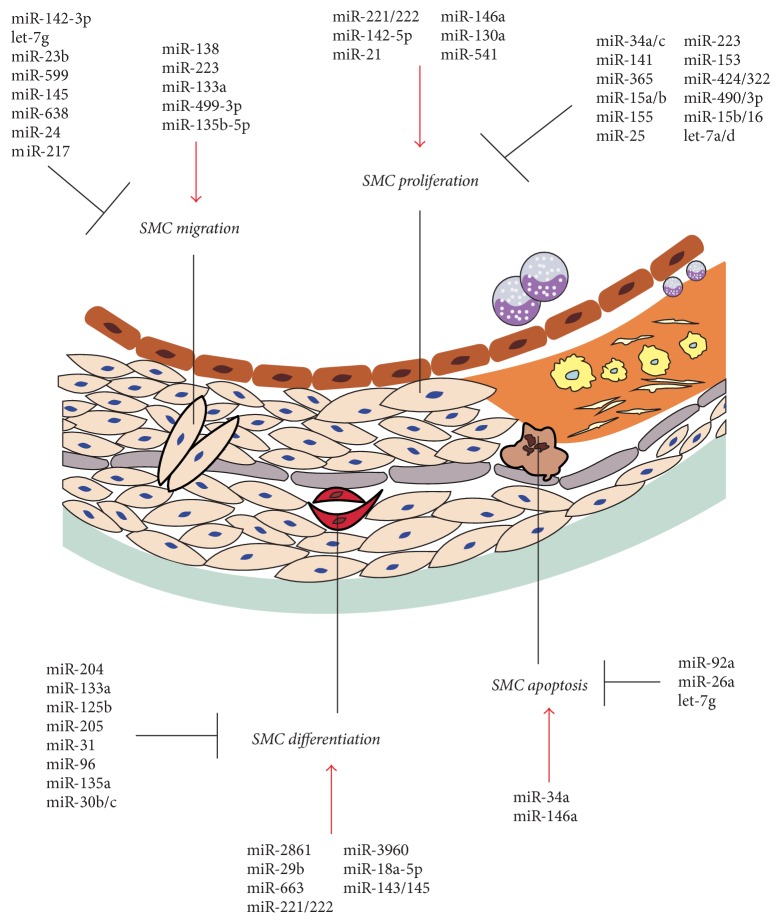
miRNAs that regulate phenotypic switching in VSMCs. The picture shows a variety of miRNAs that are involved in regulating the proliferation, migration, apoptosis, and differentiation of VSMCs. The red arrows indicate stimulatory effects, while the black arrows indicate inhibitory effects.

VSMCs, the predominant cells in the tunica media of arteries, are highly specialized cells that represent the main contributor to vessel wall formation and vascular tension maintenance. The predominant phenotype of VSMCs is quiescent/contractile with nonmigratory and nonproliferative in periods of health. However, with the progress of aging and in response to various pathological stimuli, VSMCs deviate from their physiological state and switch to a proliferative, migratory, apoptotic, and differentiation phenotype, which is called phenotypic modulation or switching [73]. Recently, emerging evidence has revealed that miRNAs are involved in vascular disease through the regulation of VSMC migration, proliferation, differentiation, and apoptosis [74–77]. Next, we will summarize the current knowledge on the role of miRNAs in the regulation of VSMCs functions, including proliferation, migration, apoptosis, and differentiation (Figure 2).

#### 2.2.1. miRNAs and VSMCs Apoptosis and Senescence

Apoptosis and senescence of VSMCs have been identified as important processes in a variety of human vascular diseases, such as atherosclerosis [78, 79]. ox-LDL plays an important role in atherogenesis. Studies have shown that hsa-let-7g can inhibit ox-LDL uptake and reduce apoptosis in SMCs by the downregulation of cytochrome C and Smac/Diablo and upregulation of Bcl-2 expression [74, 80]. In addition, miR-34a, an aging-associated miRNA, can promote VSMCs senescence and inflammation through SIRT1 downregulation and senescence-associated secretory phenotype factor induction, respectively [76]. Moreover, miR-92a overexpression inhibits H_2_O_2_-induced VSMCs apoptosis and senescence by suppressing both mitogen-activated protein kinase 4 (MKK4) and JNK1 pathways [81]. Another miRNA, miRNA-146a was found to induce VSMC apoptosis via activation of the NF-*κ*B signaling pathway [82].

#### 2.2.2. miRNAs and VSMCs Proliferation and Migration

In the native vessel, VSMCs are maintained in a quiescent/contractile, nonmigratory and nonproliferative state. In response to vascular or mechanical injury, VSMCs switch to the dedifferentiated/synthetic phenotype and increase their ability to migrate to the intima space, proliferate, and produce the ECM, which contributes to the development of atherosclerosis. Therefore, the proliferation and migration of VSMCs are closely associated and together play a central role in the growth of atherosclerotic lesions. An increasing number of studies have demonstrated that miRNAs play an important role in the regulation of VSMC proliferation and migration [83–85].

*(1) miRNAs That Promote the Proliferation and Migration of VSMCs.* Some miRNAs have been found to promote the proliferation and migration of VSMCs. miR-21 is one of the most abundant miRNAs in the vascular wall following balloon injury; it can enhance VSMCs migration and proliferation caused by TSP-1 [86] and stimulate the proliferation and migration of VSMCs through the suppression of c-Ski [83]. c-Ski is a molecule that is expressed in VSMCs to suppress VSMC stimulation and intimal hyperplasia in a rat balloon injury model [87]. Therefore, in cultured human VSMCs, low expression of miR-21 significantly inhibits cell proliferation and migration by targeting different genes [88, 89]. miR-146a, a novel regulator of VSMC fate, promotes VSMCs proliferation and migration by targeting Krüppel-like factor 4 (KLF4) mRNA [90, 91]. Moreover, miR-146a and miR-21 were significantly upregulated in atherosclerotic plaques and cooperated to accelerate VSMC growth and cell cycle progression by targeting Notch2 and Jag1 [92]. Interestingly, miR-221/222, contrary to its effects of antiproliferation, antimigration, and proapoptosis in ECs, had the effects of proproliferation, promigration, and antiapoptosis in VSMCs. The different expression profiles of the target genes p27(Kip1), p57(Kip2), and c-kit between the two cell types might be related to the opposite effects [85, 93].

Diabetic VSMCs exhibit significantly increased rates of proliferation and migration, which is the most common pathological change in atherosclerosis. miR-138 promotes the proliferation and migration of VSMCs in db/db mice by suppressing the expression of SIRT1 [94], and miR-133a serves as a stimulatory factor for IGF-1R expression by prolonging the half-life of IGF-1R mRNA and promoting IGF-1-induced VSMC proliferation in murine atherosclerosis [95]. Therefore, identification of the miR-138 and miR-133a-IGF-1R pathways might provide insight into the design of an efficient therapeutic approach to suppress atherosclerosis. In addition, other miRNAs, including miR-130a [96], miR-135b-5p and miR-499a-3p [97], miR-142-5p [98], miR-223 [99], miR-155 [100], and miR-541 [101], could promote VSMC proliferation and migration by regulating their own target genes.

*(2) miRNAs That Inhibit the Proliferation and Migration of VSMCs.* Other miRNAs have been reported to inhibit the proliferation and migration of VSMCs. By preventing VSMC proliferation, neointimal progression in atherosclerosis may be controlled. The let-7 family plays an important role in VSMC function. Let-7a decreased the proliferation of cultured VSMCs by reducing the expression of c-Myc and KRAS and could prevent intimal hyperplasia in an experimental vein graft model [102]. Overexpression of let-7d reduces VSMC growth by targeting KRAS [103]. Lower levels of let-7g have been observed both in subjects with hypercholesterolemia and in mice fed a high-fat diet. The transfection of let-7g into VSMCs has been shown to significantly inhibit VSMCs proliferation and migration induced by ox-LDL by targeting LOX-1 [104]. Moreover, both miR-141 and miR-490-3p could inhibit ox-LDL-induced VSMC proliferation through targeting of PAPP-*α* [105, 106].

Diabetes is another common age-related disease; VSMCs play a key role in the progress of diabetic atherosclerosis. miR-24 could inhibit high-glucose-induced VSMCs proliferation and migration by targeting high mobility group box-1 (HMGB1) [107]. Meanwhile, the G1/S transition activated by platelet-derived growth factor-BB (PDGF-BB) could be blocked by miR-365 [108] and miR-15b [109]. Additionally, miR-638 also mediated inhibitory effects on PDGF-induced cell proliferation and migration in human aortic SMCs by targeting the NOR1/cyclin D pathway [110]. Qian et al. reported that upregulating miR-542-3p in old VSMCs could inhibit VSMCs proliferation by directly targeting spleen tyrosine kinase. This downregulation of miR-542-3p may explain age-related neointimal hyperplasia in rats [111].

As mentioned in the miRNAs participate in the function of ECs, miR-34a and miR-34c inhibited VSMCs proliferation and migration through the modulation of Notch gene and stem cell factor (SCF) expression, respectively, which contributed to reducing neointimal hyperplasia [75, 112]. Specifically, overexpression of miR-223 and miR-153 inhibited stretch stress-enhanced VSMCs proliferation via activation of the insulin-like growth factor-1 receptor and PI3K-AKT signaling pathway [113]. Besides, miR-155 and miR-217 would inhibit angiotensin II and homocysteine-induced VSMC proliferation and migration [114, 115]. In contrast, miR-132 and miR-125b could block VSMC proliferation and neointimal hyperplasia in atherosclerosis [116, 117]. Other miRNAs that inhibit VSMCs proliferation and migration include miR-142-3p [118], miR-145 [119], miR-599 [120], miR-25 [121], miR-23b [122], miR-15b/16 [123], and miR-29b [124].

#### 2.2.3. miRNAs and VSMCs Differentiation and Calcification

Vascular calcification is a highly prevalent phenomenon among the elder population and is identified frequently in patients with atherosclerosis, diabetes mellitus, and chronic kidney disease (CKD) [125–127]. One component of the vascular calcification process involves the reprogramming and transdifferentiation of VSMCs to osteoblast-like cells [128–131]. These osteoblast-like SMCs generate and release calcifying matrix vesicles that are another essential factor involved in vascular calcification [132–134]. As the process of vascular calcification is tightly regulated and involves the genetic reprogramming of VSMCs, it is not surprising that there is accumulating evidence to support an integral role for miRNAs in this process [135–138].

The transdifferentiation of VSMCs to osteoblast-like cells that from the bone matrix is a recognized contributor to vascular calcification. Our previous studies had demonstrated that miR-204 and miR-133a could reduce osteoblastic differentiation of VSMCs induced by *β*-glycerophosphoric acid (*β*-GP) via targeting runt-related transcription factor 2 (Runx2) [9, 10]. Wen et al. also identified that overexpression of miR-125b could inhibit *β*-GP-induced osteogenic marker expression and the calcification of VSMCs. Moreover, miR-125b targeted Ets1 and regulated its protein expression in VSMCs [138]. Furthermore, endogenous miR-205 inhibits the differentiation of HA-VASMCs into osteoblast-like cells by targeting Runx2 and Smad1, as evidenced by a decrease in ALP activity, osteocalcin secretion, and Runx2 expression [139], whereas miR-2861 and miR-3960 in VSMCs enhance *β*-GP-induced osteogenic transdifferentiation of VSMCs by targeting histone deacetylase 5 or Homeobox A2, respectively, resulting in increased Runx2 protein production [140]. The overexpression of miR-29b promoted Pi-induced VSMC calcification; thus, it plays an important role in the progression of vascular calcification via osteoblastic differentiation in VSMCs [136].

Many other miRNAs could be important biomarkers of diseases through modulation of the VSMCs phenotype. CREG and VSMCs differentiation marker gene expression levels were shown to be suppressed by miR-31 [141]. BMP signaling downregulates the transcription of miR-96, which in turn leads to upregulation of Tribbles-like protein 3 (Trb3), an essential positive regulator of the BMP signaling pathway, and promotes the contractile phenotype in VSMCs [142]. When overexpression of miR-663 and miR-18a-5p promotes VSMCs differentiation markers, SM *α*-actin and SM22*α* are involved in VSMCs differentiation by targeting JunB/myosin light chain 9 and syndecan-4 expression, respectively [143, 144]. mR145 acts to suppress TGF*β*-dependent ECM accumulation and fibrosis, while promoting TGF*β*-induced VSMCs differentiation [145]. At the same time, miR-145 and miR-143 cooperatively target a network of transcription factors, including KLF4, myocardin, and ELK-1 (ELK1, member of the ETS oncogene family), to promote differentiation and repress the proliferation of SMC [146], and VSMC differentiation marker genes such as SM-actin, calponin, and SM-MHC are upregulated by premiR-145 and miR-145 mediated phenotypic modulation of VSMCs through its target gene KLF5 and its downstream signaling molecule, myocardin [147].

Other miRNAs are involved in modulating the differentiation of VSMCs. For example, miR-135a acts as a potential osteogenic differentiation suppressor in senescent VSMCs by targeting both KLF4 and STAT3 [137]. Increased calcium deposition was observed in the combined treatment with mimics of miR-221 and miR-222 [135]. In VSMCs, miR-762, miR-714, and miR-712 were involved in calcification by disrupting Ca2t efflux proteins [148]. Additionally, BMP-2 downregulates miR-30b and miR-30c to increase Runx2 expression in VSMCs and promote mineralization and VSMCs calcification [149].

Several important miRNAs could regulate various functions of VSMCs. For instance, transfection of let-7g into VSMCs has been shown to significantly inhibit VSMCs proliferation and migration induced by ox-LDL by targeting LOX1 [104]. Moreover, let-7g can inhibit ox-LDL uptake and reduce apoptosis in SMCs via downregulation of cytochrome C [74]. In addition, miR-221/222 not only inhibits the differentiation of VSMCs but also promotes their proliferation and migration [85, 135]. In the process of regulating VSMCs function, the SIRT1 gene also has important effects on VSMCs, as it does on ECs. For example, miR-34a can promote VSMCs apoptosis by modulating the expression of SIRT1 [76], while miR-138 can promote the proliferation and migration of VSMCs by inhibiting the expression of SIRT1 [94]. Just like the SIRT1 gene, KLF4, a member of the family of evolutionarily conserved zinc finger-containing transcription factors, could be taken as a regulatory target of different miRNAs to regulate the proliferation, migration and differentiation of VSMCs. For example, miR-146a could promote VSMC proliferation and migration by targeting KLF4 [91], whereas miR-15a acts as a direct transcriptional target of KLF4 that mediates the antiproliferative and antiangiogenic actions of VSMCs [150]. Meanwhile, miR-143 and miR-145 cooperatively target a network of KLF4 to promote differentiation and repress the proliferation of VSMCs [146].

## 3. How to Analyze the Role of miRNAs in Cells

The effects of miRNAs on the regulation of ECs and VSMCs are not a set of isolated processes; many miRNAs participate in modulating the function of both ECs and VSMCs including miR-221/222, miR-34a, miR-21, miR-217, miR-132, and the let-7 family. However, even the same miRNAs might have different effects on ECs and VSMCs. For example, miR-21 can enhance the rapamycin-induced inhibition of endothelial proliferation by targeting RhoB [37]. However, it can stimulate VSMCs proliferation and migration through suppression of c-Ski, and the low expression of miR-21 significantly inhibits cell proliferation and migration by targeting different genes [88, 89]. miRNAs, however, could also have a similar influence on ECs and VSMCs. For instance, let-7g negatively regulated apoptosis in the ECs by targeting caspase-3 expression [21]. Meanwhile, let-7g could inhibit SMC apoptosis by downregulating cytochrome C [74]. On the one hand, miR-221/222 could partly alleviate apoptotic cell death mediated by ox-LDL through the suppression of Ets-1 and its downstream target, p21 [18]. On the other hand, miR-221/222 also had antiapoptosis effects in VSMCs [85]. In addition, miR-34a expression is increased in senescent HUVECs and induces HUVEC senescence through the suppression of SIRT1 [29]; at the same time, it could inhibit cell proliferation and migration by targeting SIRT1 [39]. In VSMCs, miR-34a can also promote VSMCs senescence and inflammation through SIRT1 downregulation [76]. Furthermore, miR-34a inhibited VSMC proliferation and migration by modulating SCF expression [112]. The main explanation for the different roles of miRNA in ECs and VSMCs may be as follows: firstly, different cell types display their own unique characteristics and functions. Secondly, the particular structures and characteristics of different miRNAs play a key role in the functions of cells. Thirdly, the differing results may be related to the detailed conditions of the experiment. Finally, the target genes selected in the experiment may also influence miRNA functions. Different target genes have different biological properties; therefore, if miRNAs targeted the same genes in ECs and VSMCs, they will have similar effects. For example, miR-34a targets SIRT1 in both ECs and VSMCs; thus miR-34a has the same inhibitory effects on senescence and proliferation in the two cell types [29, 76, 112]. Nevertheless, different miRNAs with the same target genes may also produce different effects. For example, miR-146 can downregulate SIRT1 and promote ECs senescence [30], whereas let-7g, also with SIRT1 as the target gene, has inhibitory effects on ECs senescence [33]. Therefore, it is necessary to assess the specific environment and the target genes when analyzing the role of a miRNA.

## 4. Prospective Clinical Application of miRNAs as Diagnostic and Therapeutic Tool for Vascular Diseases

miRNAs have become one of the most important gene regulators involved in almost all types of cellular processes, including vascular cell differentiation, migration, proliferation, senescence, and apoptosis. miRNAs that are detected in serum or plasma are collectively called circulating miRNAs and the source of that might be vesicles (exosomes and microparticles), proteins, or lipoprotein complexes, which might fulfill biological functions outside the cell and act as potential biomarkers for cardiovascular diseases [151]. Although various tissues such as the heart, lung, liver, and kidney contribute to the circulating miRNA pool, most of the miRNAs are derived from blood cells [152].

It is generally considered that circulating miRNAs may provide a specific signature that reflects a given disease state; thus, measurement of circulating miRNAs can serve as a diagnostic tool in cardiovascular disease. For example, Li et al. investigated the relative expression of miRNAs in intima samples of peripheral artery disease patients and found that miR-21, miR-27b, miR-130a, miR-210, and let-7f were significantly upregulated, whereas miR-221 and miR-22 were decreased. In addition, miR-27b, miR-210, and miR-130a were increased in serum samples. Such miRNAs would be regarded as biomarkers for early atherosclerosis [153]. Moreover, those miRNAs (miR-204, miR-125b, miR-205, and so on) that inhibit vascular calcification are downregulated, while other miRNAs (miR-2861, miR-390, and miR-29b) that could enhance vascular calcification are upregulated. Therefore, measuring circulating miRNAs levels might be a method to diagnose vascular calcification. So far, there are three major methods that could be applied for circulating miRNA identification and quantification. One is microarray technology, which has been utilized to provide a comprehensive miRNA expression profile. The other is real-time quantitative PCR (qRT-PCR), which is a simple tool that can efficiently determine the amount of a gene transcript in a given sample. The third one is next-generation sequencing (NGS), which provides us with an opportunity to examine all miRNA variants simultaneously, thereby helping in the identification of novel, disease-related miRNAs [154]. However, there are also some problems using these technologies to measure the circulating miRNAs. Firstly, the simplicity of this methodology can itself be problematic [155]. For example, there is no consensus as to whether plasma or serum is a more reliable substrate for measuring circulating miRNAs. Secondly, hemolysis during sample preparation, or even due to physiological processes, can also affect the levels of circulating miRNAs [156]. Moreover, antiplatelet treatment may affect circulating miRNAs in plasma and serum samples and may act as a confounding factor in case-control studies relating plasma miRNAs to cardiovascular disease [157]. Finally, different tissues could express the same miRNAs, which are all transmitted to the blood; thus, the measurement of circulating miRNAs lacks specificity. Therefore, there is a long way to go to increase the diagnostic accuracy of circulating miRNAs to diagnose cardiovascular diseases.

Specific miRNA expression can be modulated by genetic approaches including overexpression or silencing of the prospective miRNA [158]. Thus, delivery of miRNA mimics into the proper tissue can provide a therapeutic benefit by enhancing the levels of specific miRNAs whose expression is downregulated in the disease state. Chen et al. demonstrated that overexpression of miR-126 inhibits vascular ECs apoptosis through targeting of PI3K/Akt signaling [15]. Consistent with this study, adenovirus-mediated restoration of miR-145 into rat balloon-injured carotid arteries in vivo significantly inhibited neointimal lesion formation [147]. Nevertheless, for specific miRNAs that are upregulated during disease, silencing of specific miRNAs would be beneficial. Currently, modified oligonucleotides can be designed to complement either the mature miRNA or its precursors leading to the inhibition of specific miRNA [159]. Liu et al. have applied modified antisense oligonucleotides to successfully knock down miR-221 and miR-222 in cultured VSMCs and significantly inhibit cell proliferation and neointimal growth in rat balloon-injured carotid arteries [85]. However, because miRNAs are endogenous, restoration of aberrantly expressed miRNAs, both upregulated and downregulated, to physiological levels cannot be achieved without some unexpected side effects. For instance, the inhibition of a specific miRNA may be beneficial concerning atherosclerosis progression but may adversely affect other organ systems causing immunosuppression, liver damage, or even oncogenesis.

It is well known that miRNAs have an inhibitory effect in their targets mRNA transcription and consequently, on gene expression. In other words, the inhibition of miRNAs induces gene expression while the addition or enhancement of miRNAs has the opposite effect. Hence, the greatest challenge here lies in the ability to predict the exact effects of miRNA modulation in the human body. However, one miRNA can have multiple targets; for example, miR-21 can enhance the rapamycin-induced inhibition of endothelial proliferation by targeting RhoB [37]. Meanwhile, miR-21 significantly inhibited VSMC proliferation and migration by targeting tropomyosin and AP-1 [88, 89]. One gene can also be regulated by several miRNAs. For instance, miR-217 and miR-146a promote senescence with a reduction of SIRT1 in ECs [30, 31], whereas let-7g has an effect on reducing ECs senescence by increasing the SIRT1 protein [33]. Keeping this in mind, miRNA-based therapy may have both advantages and disadvantages. miRNAs that have only a single target gene should be easy to suppress using anti-miRNA technology, which represents an advantage. However, the suppression of miRNAs that have multiple target genes will affect several genes and might induce some unexpected side effects, which could be a disadvantage [160]. Therefore, although targeting miRNAs represents promising therapeutic strategies, careful monitoring and studying of these interactions is essential in order to guarantee a safe application in humans.

## 5. Conclusion

Aging and its associated diseases remain a huge burden especially within the next decades; research efforts are increasing to identify the underlying molecular mechanisms and especially innovative treatment approaches to diseases closely associated with aging. To date, accumulating evidence has revealed that miRNAs are becoming one of the most fascinating areas of biology and play a crucial role in regulating aging processes in animal models and humans. The relative role of different miRNAs in vascular biology as direct or indirect posttranscriptional regulators of genes implicated in structural remodeling, inflammation, angiogenesis, atherosclerosis, in-stent restenosis, and thrombosis indicates that miRNAs may serve as promising drug targets or potential biomarkers in the prevention and management of vascular disorders. In this review, we have summarized the roles of miRNAs in the regulation of vascular aging, especially with respect to EC and VSMC functions, including differentiation, proliferation, migration, senescence, and apoptosis, all of which play critical roles in the pathogenesis of vascular aging. With rigorous fundamental and clinical studies, a clearer understanding of miRNAs as biomarkers and targets for cardiovascular disease will provide new insight into vascular aging and aging-related diseases.

## Figures and Tables

**Table 1:** miRNAs implicated in ECs functions.

ECs	miRNAs	Targets	Reference
*Cellular apoptosis*			

Inhibit	miR-21	PTEN	[13]
	miR-26a	TRPC6	[14]
	miR-126	PI3K/Akt	[15]
	miR-130a	MAP3K12	[16]
	miR-155	Unknown	[17]
	miR-221/222	Ets-1/caspase-7	[18]
	miR-495	CCL2	[19]
	let-7a/b	LOX-1	[20]
	let-7g	Caspase-3	[21]

Promote	miR-19b	Apaf1	[22]
	miR-132	SIRT1	[23]
	miR-200c	ZEB1	[24]
	miR-223	IGF-1	[25]
	miR-365	Bcl-2	[26]
	miR-US25-1	BRCC 3	[27]

*Cellular senescence*			

Promote	miR- 22	Vasohibin-1	[28]
	miR-34a	SIRT1	[29]
	miR-146a	SIRT1	[30]
	miR-200c	ZEB1	[24]
	miR-217	SIRT1	[31]

Inhibit	miR-92a	Unknown	[32]
	let-7g	SIRT1	[33]

*Cellular proliferation*			

Promote	miR-29a	HBP1	[34]
	miR-126-5p	Dlk1	[35]
	miR-487b	THBS1	[36]
	miR-495	CCL2	[19]

Inhibit	miR-21	RhoB	[37]
	miR-24	Sp1	[38]
	miR-34a	SIRT1	[29]
	miR-92a	SIRT1	[39]
	miR-101	mTOR	[40]
	miR-125a	Bcl-2	[41]

*Cellular angiogenesis*			

Proangiogenesis	miR-92a	PTEN	[42]
	miR-126	Spred-1	[43]

Antiangiogenesis	miR-15a	FGF2 and VEGF	[44]
	miR-20a	MKK3	[45]
	miR-21	RhoB	[46]
	miR-351	STAT3	[47]
	miR-214,	XBP1	[48]
	miR-223	*β*1 integrin	[49]
	miR-221/222	c-Kit	[50]
	miR-106	STAT3	[51]

*Cellular inflammation*			

Promote	miR-21	PPAR*α*	[52]
	miR-92a	SOCS5	[53]

Inhibit	miR-30-5p	Ang2	[54]
	miR-126	VCAM-1	[55]
	miR-155	Ang II type 1 receptor	[56]
	miR-181b	NF-kB	[57]
	miR-663	SLC7A5 and NAV2	[58]
	let-7g	TGF-*β*	[33]

EC, endothelial cell; PTEN, phosphatase and TENsin homologue; PI3K: phosphatidylinositol 3-kinase; TRPC6: transient receptor potential canonical 6; MAPK: mitogen-activated protein kinase; Ets-1: E26 transformation-specific 1; CCL2: C–C motif chemokine 2; LOX-1: lectin-like low-density lipoprotein receptor 1; Apaf-1: apoptotic protease-activating factor; SIRT1: silent information regulator 1; ZEB1: zinc finger E-box-binding homeobox 1; IGF-1: insulin-like growth factor-1; BRCC 3: BRCA1-BRCA2-containing complex; HBP1: HMG box-containing protein-1; Dlk1: delta-like 1 homologue; THBS1: thrombospondin 1; Sp1: specificity protein 1; mTOR: mammalian target of rapamycin; FGF2: fibroblast growth factor; VEGF: vascular endothelial growth factor; MKK3: mitogen-activated protein kinase kinases 3; STAT3: signal transducer and activator of transcription 3; XBP-1, a key unfolded protein response transcription factor; PPAR*α*: peroxisome proliferator-activated receptor-*α*; SOCS5: suppressor of cytokine signaling 5; VCAM-1: vascular cell adhesion molecule 1; NF-kB: nuclear factor-kappa B; TGF-*β*: tumor growth factor-*β*.
